# Examination of the Psychometric Properties of the Observable Social Cognition Rating Scale (OSCARS) in Arabic-Speaking Patients with Schizophrenia

**DOI:** 10.3390/brainsci15090902

**Published:** 2025-08-22

**Authors:** Feten Fekih-Romdhane, Georges Kerbage, Nagham Hachem, Michelle El Murr, Georges Haddad, Rony Abou Khalil, Frederic Harb, Elissar El Hayek, Souheil Hallit

**Affiliations:** 1 The Tunisian Center of Early Intervention in Psychosis, Department of Psychiatry “Ibn Omrane”, Razi Hospital, Manouba 2010, Tunisia; 2 Faculty of Medicine of Tunis, Tunis El Manar University, Tunis 1068, Tunisia; 3 School of Medicine and Medical Sciences, Holy Spirit University of Kaslik, Jounieh P.O. Box 446, Lebanonsouheilhallit@usek.edu.lb (S.H.); 4 School of Arts and Science, Holy Spirit University of Kaslik, Jounieh P.O. Box 446, Lebanon; 5 Psychiatry Department, Psychiatric Hospital of the Cross, Jal Eddib 1106, Lebanon; 6 Faculty of Medicine and Medical Sciences, University of Balamand, Kalhat P.O. Box 100, Lebanon; 7 Department of Psychology, College of Humanities, Effat University, Jeddah 21478, Saudi Arabia; 8 Applied Science Research Center, Applied Science Private University, Amman 11931, Jordan

**Keywords:** social cognition, OSCARS, schizophrenia, psychometric properties, Arabic

## Abstract

**Background/Objectives**: No Arabic-language version of the Observable Social Cognition Rating Scale (OSCARS) is available that allows to properly and specifically assess social cognition (SC) in Arabic-speaking populations. This study aimed to examine the preliminary psychometric characteristics of the Arabic translated version of the OSCARS, including factor structure, reliability, concurrent validity, and measurement invariance across sex. **Methods**: This cross-sectional study has been conducted during February and March 2024 and included 113 chronic, remitted, and clinically stable patients with schizophrenia. **Results**: The originally proposed two-factor model (Social Cognitive Bias and Social Cognitive Ability) showed acceptable model fit after removal of two items that yielded low factor loadings (items 2 and 3). Total and factor scores showed good internal consistency, with Cronbach’s alpha of 0.85–0.94. Measurement invariance was established across sex groups at the configural, metric, and scalar levels. No significant differences emerged between male and female patients for latent mean scores of the OSCARS. Finally, concurrent validity was supported by appropriate patterns of correlations with functioning, recovery, and emotional intelligence measures. **Conclusions**: The Arabic OSCARS stands out as a brief, valid, reliable, and comprehensive assessment tool to evaluate SC in Arabic-speaking patients with schizophrenia based on the perspectives of interviewers. Offering this measure to clinicians and researchers who work in Arab settings may close the existing gap in the assessment of SC in schizophrenia. Due to its easy and fast application, the Arabic OSCARS is believed to be highly valuable in clinical and research practices.

## 1. Introduction

Social cognition (SC) encompasses an array of cognitive processes that underscore interpersonal interactions, including the way how individuals perceive, interpret, make sense of, and respond to the intentions, dispositions, and behaviors of others in the social world [1,2]. Meta-analyses show SC impairments in schizophrenia [3,4,5,6,7], a chronic disabling disease. SC is linked to social functioning, showing small-to-medium effects and partially mediating the relationship between neurocognition and functional outcome [8]. Research has even claimed that SC has closer connections with functional outcomes than neurocognition [9]. At the same time, these outcome changes were shown to be closely related to the ethnicity of participants, even when patients with a diagnosis of schizophrenia live in the same country [10,11]. Not only ethnic and cultural diversity, but also spirituality and religion, seem to play a crucial role in exposure to environmental risk factors, adherence, treatment maintenance, and overall outcome of the disease [12].

A meta-analysis showed that patients with schizophrenia performed mildly to severely worse than controls across a variety of SC domains, including ToM, social perception, emotion perception, and emotion processing [6]. Consequently, the crucial relevance of studying SC processes in schizophrenia has been highlighted by several researchers as a way to improve knowledge about the etiology of the disease [13,14]. In addition, social cognition has potential clinical relevance in schizophrenia, affecting functional outcomes and subsequently the overall recovery process [15]. This has led to a growing interest in development of intervention strategies targeting SC [16,17], which produced significant improvements in SC abilities and were widely disseminated across the world. Interestingly, SC was found to be under the influence of culture and ethnicity, which may pose an important challenge to its assessment in patients with schizophrenia, given the shared variance between sociocultural factors and psychopathology [18].

### 1.1. Measurement Instruments of SC in Schizophrenia

There are several instruments currently available to assess SC in persons with schizophrenia, but most of them have multiple limitations that hinder our understanding of the construct and limit its usefulness as a viable target for intervention. Among the limitations are uncertainties on the exact construct assessed with some of the measures. For example, the Mayer–Salovey–Caruso Emotional Intelligence Test [19] was initially intended to measure emotional intelligence abilities. The task requires respondents to rate how effective they can be in managing their emotions in social scenarios. It, thus, assesses some capacity to perceive emotions, but it does not directly assess facial emotion perception with the use of photos. Another measure, i.e., the Reading the Mind in the Eyes Test [20], which was originally designed to assess theory of mind, seemed to assess empathy or emotion recognition capacity rather than the ability to attribute mental states [4]. Researchers have also questioned the usefulness of the Attributional Style Questionnaire [21] in assessing the specific attributional styles [22]. Overall, these tools appear to measure specific sub-domains, such as only emotion perception or theory of mind, and were mostly used collectively for a complete assessment of the SC concept and its core domains. This approach might, however, pose some problems in practice [23,24]. Using several measures to assess a single (multidimensional) construct can also be time-consuming and burdening for patients, especially since previous experiences showed that patients with schizophrenia may have limited interest and motivation to complete research instruments (e.g., [25]). Another important methodological limitation to the existing measures of SC is that their psychometric qualities are often not well-established [3,23,26]. To address these gaps, Healey et al. [27] designed the Observable Social Cognition Rating Scale (OSCARS) as a single measurement instrument with a broad scope to assess SC as an umbrella concept and solid psychometric properties.

### 1.2. The OSCARS

The OSCARS assesses SC performance through eight items covering the following different SC domains: empathy, cognitive rigidity, theory of mind, jumping to conclusions, attributional style, and emotion perception. A sample item is “Recognizing other people’s emotions, particularly negative emotions (sadness, fear and anger) based on facial expression, body language and/or vocal tone and rate”. Items were scored on a 7-point Likert scale from 1 (none; e.g., “Can recognize strong, moderate and subtle expressions of emotions. She/he can be thought of as socially perceptive”) to 7 (extremely severe; e.g., “Never or does not recognize strong, moderate and subtle emotional expressions. The person must be told what emotion is being expressed”), with total scores indicating more problems with SC or a greater negative impact on everyday life [27]. The OSCARS can be flexible in different ways, either as an informant-based questionnaire (based on the informant’s knowledge of and regular interaction with the individual in everyday situations), as a semi-structured interview [27], or as a self-administered questionnaire [28].

It was firstly validated in the United States (US), in a sample of 62 outpatients with schizophrenia spectrum disorders and 50 non-psychiatric controls, where it showed evidence of construct and convergent validity, adequate internal consistency, good test-retest reliability, and correlated significantly with different functional outcome measures [27]. In this study, the OSCARS yielded a two-factor model. The first factor was labelled “social cognitive bias”, and encompasses items on attributional style (item 2), jumping to conclusions (item 3), and cognitive rigidity (items 4 and 5). The second factor, named as “social cognitive ability”, is composed of items referring to emotional perception (item 1) and theory of mind (items 6, 7, and 8). Item 6 (probing theory of mind) did not clearly load on either factor; thus, the developers chose to include it on the second factor with other items assessing theory of mind [27].

The psychometric properties of the OSCARS as both a self- and informant-reported scale were later verified and confirmed in a larger multisite research in the US, including 382 patients with schizophrenia spectrum disorders and 289 healthy controls [28]. In addition, other linguistic validations of the OSCARS, including the Spanish [29], Turkish [30], and Persian [31] versions, demonstrated similar psychometric properties to the original version. The OSCARS was also translated to the Dutch [32] and Japanese [33] languages, without examination of its psychometric properties. However, no Arabic-language version of the OSCARS has been made available that allows us to properly and specifically assess the SC construct in Arabic-speaking populations and inform the development and evaluation of strategies aimed at improving clinical and functional outcomes. In addition, only scant research has been performed on SC in schizophrenia in the Arab context.

### 1.3. Rationale of the Present Study

Based on the above literature and observations, the OSCARS was selected for translation and validation in the Arabic language as an observer-report measure able to cover multiple domains of SC relevant to individuals with schizophrenia. This step is crucial to determine whether the theoretical conceptualization of the OSCARS holds up cross-culturally and to attest to its potential use in SC research and applications. Indeed, cultural factors have proven to influence social cognitive performance and are likely to affect the development of various SC domains [18,34]. A recent meta-analysis of SC deficits in schizophrenia across different regions of the world found that the magnitude of deficits in the SC domain “hostile attributional style” was more pronounced in European and North American samples compared to Asian samples [7]. Therefore, this study aimed to examine the preliminary psychometric characteristics of the Arabic translated version of the OSCARS, including factor structure, reliability, concurrent validity, and measurement invariance across sex. It is hypothesized that the Arabic version of the OSCARS will replicate the two-factor solution proposed in the original study and will have adequate internal consistency reliability (Cronbach’s alpha coefficients > 0.70). Based on the previous literature, it is also expected that the scale in its Arabic version will correlate significantly with clinically related constructs: functioning, recovery, and emotional intelligence. Indeed, measures of functional outcome and of the emotional component of SC have been considered in previous validation studies of the OSCARS (e.g., [27,28]), where the scale consistently demonstrated positive correlations with these related constructs, establishing its convergent and concurrent validity.

## 2. Materials and Methods

### 2.1. Sample and Procedure

The study was conducted using a cross-sectional design between February and March 2024. The target population consisted of adult inpatients with schizophrenia who were admitted to a long-stay accommodation in the Psychiatric Hospital of the Cross, Jal Eddib, Lebanon. A total of 133 Lebanese inpatients with schizophrenia were approached according to a list provided by the hospital administration. Among them, 11 women and 9 men declined to take part. Of the 20 patients ultimately excluded from the study, two were unable to finish the second section due to isolation protocols, one was prevented from doing so because of work commitments, and seventeen did not provide consent, thereby opting out of both sections of the questionnaire. Participation was voluntary, with no financial or other incentives offered, and all participants were informed of their right to withdraw at any point.

Semi-structured individual interviews were undertaken with each participant who agreed to be interviewed on the ward in a single side-room at a time arranged in advance. The interviews were administered by trained students from the Faculties of Sciences and Medical Sciences, who received instruction from a psychologist and a researcher on how to conduct them. Each session lasted for around 30 min. Demographic and clinical information was collected from patients themselves and from their medical records. The following inclusion criteria were applied: “(1) age of 18 years and over, (2) with a schizophrenia disorder diagnosis following the DSM-5 criteria [35], (3) at chronic stage of the disease, defined as with more than 1 year of illness duration [36], and institutionalized in the above-mentioned long-stay hospital for more than one year [37,38]”, (4) remitted and clinically stable, as defined by Fleischhacker et al. [39]: patients “were required to be symptomatically stable, as judged by the treating physician, be receiving a stable dose of an antipsychotic drug for at least 4 weeks before the survey and be in good general physical health” [40]. A total of 185 patients were institutionalized at the hospital at the time of the survey. One hundred and nineteen patients that were approached by the research team were eligible and accepted to take part in the study. Six were excluded during the interview process because of non-cooperation. The finale total sample included 113 participants.

### 2.2. Measurements

#### 2.2.1. Sociodemographic Information

Interviewers collected information from each patient regarding age, gender, education level, duration of illness, and duration of hospitalization [40].

#### 2.2.2. The OSCARS

After obtaining permission from the original author, Professor David L. Penn, the OSCARS was rigorously translated and culturally adapted for the Arabic environment and language. The translation of the OSCARS was conducted following the Principles of Good Practice for Translation and Cultural Adaptation, using the “forward-backward-forward” technique method [41]. One Lebanese translator was in charge of the translation of the scale from English to Arabic. Then, a Lebanese psychologist who is fluent in English performed the back translation from Arabic to English. A panel of experts composed of one psychologist, two psychiatrists, the research team, and the translators compared the back-translated and original English versions. This was performed to (1) confirm the accuracy of the translation, (2) resolve any inconsistencies, and (3) ensure the conceptual consistency of the scale in both the original and Arabic settings. Following the panel review, a pilot study was conducted to validate the clarity and interpretability of the items. No subsequent changes were made to the scale. The final Arabic translation of the OSCARS can be shown in Table S1.

#### 2.2.3. The Recovery Assessment Scale (RAS-8)

This is an eight-item measure composed of two domains: goal and success orientation (5 items; e.g., “I have a desire to succeed”) and no domination by symptoms (3 items; e.g., “My symptoms interfere less and less with my life”) [42]. All items are scored on a 5-point Likert-type scale ranging from 1 (strongly disagree) to 5 (strongly agree). More elevated total scores reflect better perceived recovery (ω = 0.85/α = 0.86).

#### 2.2.4. The Global Assessment of Functioning (GAF)

This measure assesses social functioning [43], assigning a score between 0 and 100 to each patient based on the interviewer’s evaluation of the current level of impairment in educational, occupational, and/or psychosocial domains.

#### 2.2.5. The Brief Emotional Intelligence Scale (BEIS-10)

Validated in Arabic [44], this scale measures trait emotional intelligence via 10 items [45]. Each item is scored on a 5-point scale ranging from 1 (strongly agree) to 5 (strongly disagree). More elevated scores indicate higher levels of EI (ω = 0.82/α = 0.87).

### 2.3. Analytic Strategy

There were no missing responses in the dataset. We used data from the total sample to conduct a CFA using the SPSS AMOS v.29 software. We aimed to enroll a minimum of 24–160 persons based on 3–20 times the number of the scale’s variables [46]. The maximum likelihood method was used to obtain the following fit indices: root mean square error of approximation (RMSEA ≤ 0.08), standardized root mean square residual (SRMR ≤ 0.05), Tucker-Lewis Index (TLI ≥ 0.90), and Comparative Fit Index (CFI ≥ 0.90) [47]. Multivariate normality was not verified at first; therefore, we performed a non-parametric bootstrapping procedure.

To examine sex invariance of OSCARS scores, we conducted multi-group CFA [48] using the total sample. Measurement invariance was assessed at the configural, metric, and scalar levels [49]. We accepted ΔCFI ≤ 0.010 and ΔRMSEA ≤ 0.015 or ΔSRMR ≤ 0.010 as evidence of invariance [50]. Mann–Whitney test was used to compare two means.

Composite reliability was assessed using McDonald’s ω and Cronbach’s α, with values greater than 0.70 reflecting adequate composite reliability. The Spearman test was used to correlate the OSCARS scores with other continuous variables.

## 3. Results

### 3.1. Participants

The mean age of participants was 57.52 ± 10.35 years, and 63.5% were males. Other characteristics of the patients are summarized in Table 1.

### 3.2. Confirmatory Factor Analysis

**Figure 1:**
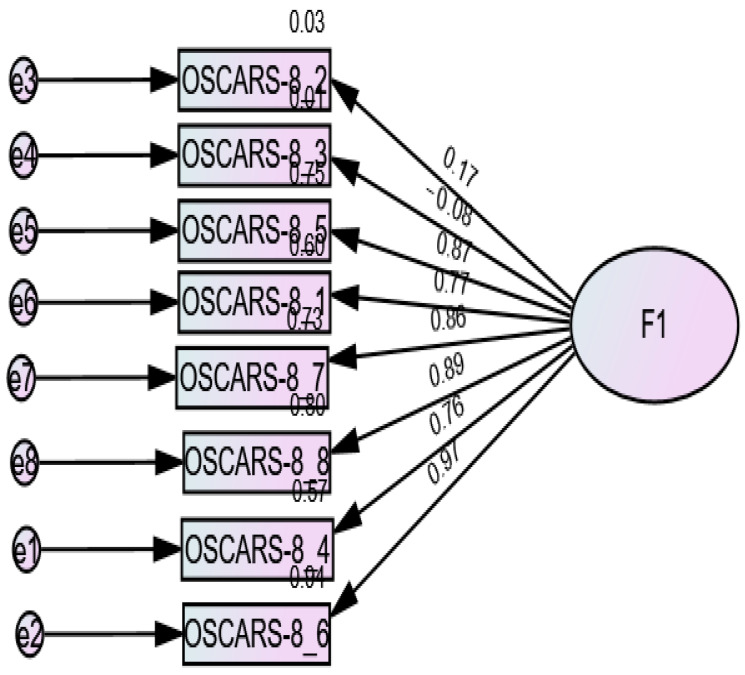
Standardized loading factors of the one-factor model (eight items) of the OSCARS.

**Figure 2:**
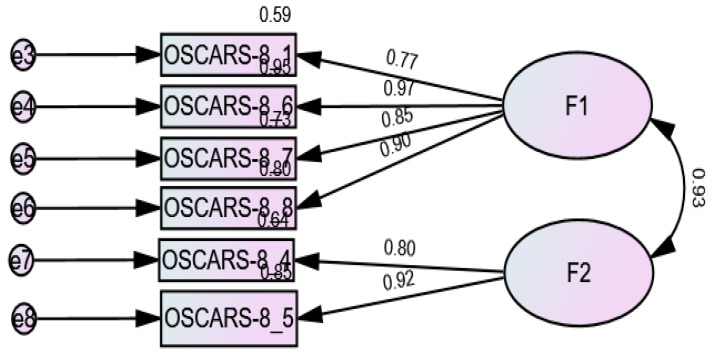
Standardized loading factors of the two-factor model of the OSCARS.

**Figure 3:**
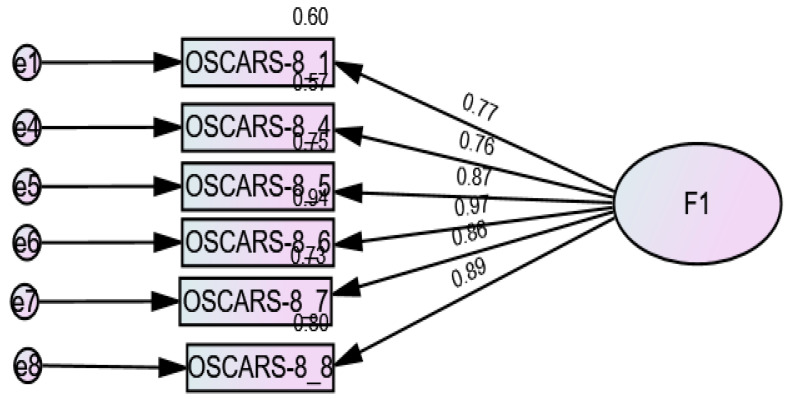
Standardized loading factors of the one-factor model (six items) of the OSCARS.

**Figure 4:**
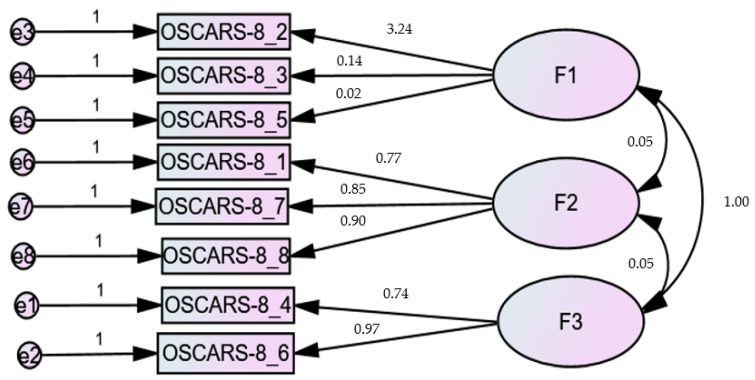
Standardized loading factors of the three-factor model of the OSCARS.

CFA indicated that the fit of the one-factor model (8 items) was modest: RMSEA = 0.157 (90% CI 0.120, 0.195), SRMR = 0.093, CFI = 0.917, TLI = 0.884 (Figure 1). The fit of the two-factor model [27,29,30] of the OSCARS scale was modest: RMSEA = 0.151 (90% CI 0.113, 0.191), SRMR = 0.092, CFI = 0.927, TLI = 0.893. However, the loading factors of items 2 and 3 were low (<0.33) and therefore removed; the CFA results of the six-item model showed the following fit indices: RMSEA = 0.106 (90% CI 0.039, 0.172), SRMR = 0.032, CFI = 0.984, TLI = 0.970 (Figure 2). The fit indices of the one-factor model (6 items) were RMSEA = 0.129 (90% CI 0.072, 0.189), SRMR = 0.034, CFI = 0.973, and TLI = 0.955 (Figure 3). The fit indices of the three-factor model [27] were poor: RMSEA = 0.301 (90% CI 0.264, 0.341), SRMR = 0.032, CFI = 0.740, TLI = 0.572 (Figure 4).

The standardized estimates of factor loadings of all models are summarized in Table 2. Composite reliability values were ω = 0.94/α = 0.94 for the total score, α = 0.85 for Factor 1 (social cognitive bias), and ω = 0.93/α = 0.93 for Factor 2 (social cognitive ability). The AVE values of the one- and two-factor models were 0.73 and 0.76, respectively, confirming convergent validity.

### 3.3. Sex Invariance

We were able to show the invariance across sex at the configural, metric, and scalar levels (Table 3). No significant difference was found between males and females in terms of OSCARS total scores (27.11 ± 8.89 vs. 23.76 ± 11.55; *t*(111) = 1.61; *p* = 0.113), social cognitive bias (8.90 ± 3.05 vs. 7.59 ± 3.80; *t*(111) = 1.90; *p* = 0.062), and social cognitive ability (18.21 ± 6.23 vs. 16.17 ± 8.11; *t*(111) = 1.39; *p* = 0.168).

### 3.4. Concurrent Validity

Higher emotional intelligence (*r* = −0.64; *p* < 0.001), global functioning (*r* = −0.51; *p* < 0.001), and recovery (*r* = −0.39; *p* < 0.001) were significantly associated with lower OSCARS scores (better social cognition) (Table 4).

### 3.5. Discriminant Validity

The square root of the AVE value was 0.85, which was higher than the correlations between the OSCARS and other measured constructs, supporting discriminant validity for the scale.

## 4. Discussion

### 4.1. Overview

SC abilities allowing to navigate social cues and behaviors are largely impaired in patients with schizophrenia, and were consistently established as important precursors of decline in everyday functioning in this population [8]. SC was therefore suggested as a valuable construct for understanding the nature and disability of schizophrenia [14]. This study sought to make available a psychometrically sound observer-based measure of SC, i.e., the OSCARS, in the Arabic language. Findings suggest that the Arabic version of the OSCARS is a short, easy-to-administer, valid, and reliable tool that is suitable for use by interviewers to assess different key domains of SC among Arabic-speaking patients with schizophrenia. In the present study, CFA supported removing both items 2 and 3, which, respectively, covers the tendency to interpret others’ actions as hostile (hostile attribution bias) and to make decisions quickly without examining evidence (jumping-to-conclusions bias), as those two items showed weak loadings (<0.33) and degraded model fit. While their removal improved model fit and internal consistency, it also reduced the scale’s coverage of specific social cognitive biases. This means the Arabic OSCARS in its six-item form provides a cleaner measure of social cognitive ability and flexibility but may underrepresent certain bias-related processes.

### 4.2. Comparison with Previous Studies

The originally proposed two-factor model (Social Cognitive Bias and Social Cognitive Ability) showed acceptable model fit in our sample of Arabic-speaking patients with schizophrenia after removal of two items (items 2 and 3), which yielded low factor loadings. The differences in item loadings between the original study and the present study might be due to cultural factors. The Arabic OSCARS total and factor scores showed good internal consistency, with Cronbach’s alpha of 0.85–0.94, agreeing with the original validation study of the OSCARS [27]. The first factor (social cognition bias) reflects SC aspects related to rigidity, while the second factor (social cognition ability) evaluates indicators of reasoning and perceptual skills that are close to the theory of mind. Using the original English-language version of the OSCARS in a large multisite sample of patients with schizophrenia spectrum disorders from the US, Halverson et al. [28] were also able to further confirm the two-factor structure of the OSCARS with a satisfactory internal consistency (alpha 0.72–0.92). Consistent with our findings, other cross-cultural adaptations of the OSCARS provided additional support to its validity and reliability as a tool to assess SC by an observer in individuals with schizophrenia. For instance, the psychometric properties of the Spanish version of the OSCARS were examined in a sample of 109 chronic patients with schizophrenia who were rated by their primary caregivers; a two-factor structure was identified, with a Cronbach’s alpha coefficient of 0.75 and 0.76 for each factor and of 0.82 for the total score [29]. The Turkish language version that was validated in 50 dyads of patients with schizophrenia and schizoaffective disorder and their relatives also generated a two-factor solution, with items 2, 3, and 4 loading on the “social cognitive bias” factor and items 1, 6, 7, and 8 loading on the “social cognitive ability” factor [30]. Item 5 loaded on both factors, but it was grouped with the first factor as in the original version. Furthermore, although the RMSEA values exceeded the recommended threshold of 0.08, RMSEA tends to be inflated in models with low degrees of freedom (df < 50) [39,40]. Given that our models have low degrees of freedom (df = 9), RMSEA may not be a reliable indicator in this case. Instead, model evaluation should rely on the other fit indices, such as CFI and SRM, which have been shown to be more stable under these conditions [40]. In our study, both CFI and SRMR values were adequate, supporting a good fit despite the elevated RMSEA. The total score of the Turkish OSCARS had an adequate internal consistency, with a Cronbach’s alpha coefficient of 0.83 [30]. The Persian version of the OSCARS was administered as both self- and teacher-report among high school students aged 14–18 years from Iran; it revealed good validity, with a Cronbach alpha of 0.60 (self-report version) and 0.65 (teacher’s version) [31].

### 4.3. Measurement Invariance Across Sex

Our study investigated and established measurement invariance of the Arabic version of the OSCARS across sex groups at the configural, metric, and scalar levels. This psychometric property allows us to ensure that a valid measurement model holds across sex and that the measure’s scores reflect the same latent construct to the same degree [49]. In other words, verifying measurement invariance across sex ascertains that differences in OSCARS latent mean scores observed between male and female patients are due to true differences, not item bias or different item utilization. In the present Arabic-speaking sample, no significant differences emerged between male and female patients for latent mean scores of the OSCARS. In line with our results, earlier studies found no [51,52,53,54] or only modest [55] sex differences in social cognitive performance in schizophrenia. It is of note, however, that measurement invariance has not been previously tested for the OSCARS for groups of male and female patients with schizophrenia, to the best of our knowledge. Therefore, any previous conclusions about cross-sex differences using the OSCARS could be misleading and should be interpreted with caution. It is highly recommended that, when using the OSCARS, information on measurement invariance be provided before drawing any firm conclusions about sex differences in SC.

### 4.4. Concurrent Validity and Clinical Correlates

Concurrent validity was supported by appropriate patterns of correlations with functioning, recovery, and emotional intelligence measures. Our findings showed a significant moderate inverse relationship between SC scores and levels of functioning and recovery of patients. Consistently, other prior research also found that higher OSCARS scores (that is, more deficit in SC) were correlated with more impaired functioning [30,56]. Results of reviews and meta-analysis indicated that SC strongly predicts functioning [9,15]. In previous research, the OSCARS showed good predictive validity in that it provides an indication of impairments in real-world functioning, suggesting the scale can be more precisely conceptualized as a measure of functioning [28]. Furthermore, strong negative correlations were found between OSCARS and BEIS-10 scores in our sample; that is, more impaired SC correlated with lower emotional intelligence. Emotional intelligence and SC are often reported interchangeably in the literature, and measures designed to assess emotional intelligence (such as the Multifactor Emotional Intelligence Scale [57] or the MSCEIT [19]) have long been recognized and used as representative tests of social cognition [58]. Later, emotional intelligence has rather been regarded as a proxy of SC, with both constructs reflecting close but not the same entity [59]. In sum, these findings support the validity of the OSCARS and further emphasize the clinical relevance and importance of assessing cognitive processes that underlie how patients with schizophrenia think about social interactions, social situations, others, and themselves.

### 4.5. Study Limitations

This study has some limitations that need to be acknowledged. The sample consisted of long-stay chronic inpatients with schizophrenia and was recruited from one hospital, which might limit the generalizability of findings to outpatients and those at earlier stages of the disease. In addition, patients were recruited using convenience sampling and cannot be considered as representative of the broader population of patients with schizophrenia. However, the sample chosen was adapted to the nature of the instrument, requiring interviewers who know the participating patients well and are in frequent close contact with them. Information and inter-rater biases are possible. In addition, studies including other psychometric tests, such as test-retest using a cohort design or inter-rater reliability, are needed. It is important to note that, given the cross-sectional design of this study, correlational analyses cannot establish directionality or causality between variables. Finally, the inter-rater reliability was not examined; therefore, the agreement and consistency between different users of the scale could not be verified. Ideally, future research should include such an analysis, either through simultaneous independent interviews or by having multiple raters score recorded interviews, to robustly evaluate agreement and reduce subjective variability. Future studies should test this psychometric property to ensure that the OSCARS is consistently applied by different raters and that findings are not influenced by the subjective interpretations of individual evaluators, lending more support to its reliability.

### 4.6. Clinical Implications and Future Research Directions

The present analyses showed similar psychometric properties to the original version in patients with schizophrenia in a new culturally and linguistically different context. In particular, results lend more support for the two-factor model of the OSCARS, endorsing its use in Arabic-speaking people diagnosed with schizophrenia. Offering the Arabic validated version of the OSCARS to clinicians and researchers who work in Arab settings may close the existing gap in the assessment of SC in schizophrenia. Beyond being a robust interview-based assessment of social cognition in schizophrenia, the OSCARS stands out from other neuropsychological tests because it is one of the few instruments that can also be used in a self-report format with the targeted population [28]. Due to its easy and fast application, the Arabic OSCARS is believed to be highly valuable in clinical and research practices, particularly as the necessity of using brief, rapidly administered tools has been highlighted by clinicians and researchers [25,60]. Overall, findings support that the Arabic OSCARS has clinical potential in assessing SC, which could provide, in turn, important clues to the clinician on patients’ clinical and functional outcomes. At this stage, it is proposed that the Arabic version of the OSCARS can be implemented on an interview basis as an adequate measurement instrument for assessing and monitoring SC in schizophrenia.

Finally, some research directions can be inferred for future research. Although the external evaluation from an observer (interviewer or informant) can diminish the impact of insight, further studies are warranted to examine the psychometric properties of the OSCARS when administered as an informant- (e.g., primary caregiver) and self-report measure and compare them with the OSCARS interviewer report. In addition, future studies still need to verify the test-retest reliability of the Arabic OSCARS in order to confirm the stability of the measure at different time points. In addition, additional research may consider the comparison of psychometric properties of the Arabic OSCARS in patients with schizophrenia to those in a sample of healthy controls to examine the scale’s ability to clinically discriminate between clinical and non-clinical populations (schizophrenia vs. controls). Moreover, the present findings could help clinicians use the Arabic OSCARS not only for assessment but also to guide practical decisions in care. For example, identifying specific social cognition difficulties may help in choosing appropriate psychosocial or rehabilitation programs, setting individualized therapy goals, and tracking patient progress over time.

## 5. Conclusions

In conclusion, findings support the validity, reliability, and measurement invariance across sex groups of the Arabic-language version of the OSCARS. The Arabic OSCARS stands out as a brief, practical, and comprehensive assessment tool to evaluate SC in Arabic-speaking patients with schizophrenia based on the perspectives of interviewers. To gather information about real-world SC deficits and improve clinical and functional outcomes of this patient population, clinicians should consider using this tool in routine clinical practice. Researchers are encouraged to extend the investigation of the psychometric properties of the Arabic OSCARS in other populations, settings, and cultural contexts, and to use it in future studies.

## Figures and Tables

**Table 1:** Sociodemographic and other characteristics of the patients (*n* = 113).

Variables	*n* (%) or Mean ± SD
Gender	
Male	72 (63.7%)
Female	41 (36.3%)
Education	
Primary	27 (23.9%)
Complementary	42 (37.2%)
Secondary	30 (26.5%)
University	14 (12.4%)
Age (years)	57.52 ± 10.35
Duration of hospitalization (years)	3.69 ± 4.82
Duration of illness (years)	32.04 ± 11.59
Emotional intelligence	37.93 ± 8.42
Global functioning	71.13 ± 25.50
Recovery	24.91 ± 6.40

**Table 2:** Standardized loading factors of the all models of the OSCARS scale in Arabic.

	One Factor Eight Items	Two Factors		One Factor Six Items	Three Factors		
		Factor 1: “Social Cognitive Bias”	Factor 2: “Social Cognitive Ability”		Factor 1: “Social Cognitive Bias”	Factor 2: “Social Cognitive Ability”	Factor 3: “Social Cognitive Flexibility”
Item 1. Recognizing other people’s emotions based on facial expression, body language and/or vocal tone.	0.77		0.77	0.77		0.77	
Item 2. Interpreting social interactions in a malevolent, hostile manner.	0.17	-		-	3.24		
Item 3. Making decisions quickly without examining other evidence.	−0.08	-		-	0.14		
Item 4. Being flexible in interpreting social situations.	0.76	0.80		0.76			0.74
Item 5. Changing or correcting their interpretation of social interactions when wrong.	0.87	0.92		0.87	0.02		
Item 6. Understanding subtle jokes, sarcasm and insults in conversation.	0.97		0.97	0.97			0.97
Item 7. Seeing things from the perspective of others.	0.86		0.85	0.86		0.85	
Item 8. Understanding subtle social cues, hints and indirect requests.	0.89		0.90	0.89		0.90	

**Table 3:** Measurement Invariance of the OSCARS scores across gender in the total sample.

Model	CFI	RMSEA	SRMR	Model Comparison	ΔCFI	ΔRMSEA	ΔSRMR
Configural	0.928	0.156	0.064				
Metric	0.915	0.149	0.070	Configural vs. metric	0.013	0.007	0.006
Scalar	0.902	0.146	0.070	Metric vs. scalar	0.013	0.003	<0.001

Note. CFI = comparative fit index; RMSEA = root mean square error of approximation; SRMR = standardized root mean square residual.

**Table 4:** Correlation matrix of scores.

	1	2	3
1. OSCARS-6 items	1		
2. Emotional intelligence	−0.64 ***	1	
3. Global functioning	−0.51 ***	0.39 ***	1
4. Recovery	−0.39 ***	0.38 ***	0.41 ***

*** *p* < 0.001. Numbers in the table refer to Spearman correlation coefficients (rho).

## Data Availability

All data generated or analyzed during this study are not publicly available due to the restrictions from the ethics committee. Reasonable requests can be addressed to the corresponding author (SH).

## Supplementary Materials

The following supporting information can be downloaded at: https://www.mdpi.com/article/10.3390/brainsci15090902/s1, Table S1. The Arabic-language items of the Observable Social Cognition Rating Scale (OSCARS).

## Abbreviations

The following abbreviations are used in this manuscript:

OSCARS	The Observable Social Cognition Rating Scale
SC	Social Cognition
ToM	Theory of Mind
