# Supplementary material for: Examination of the Psychometric Properties of the Observable Social Cognition Rating Scale (OSCARS) in Arabic-Speaking Patients with Schizophrenia
Source: Brain Sci. 2025 Aug 22;15(9):902. doi: 10.3390/brainsci15090902 (PMC12468383; doi:10.3390/brainsci15090902)
Supplement: Supplementary file 1 [file brainsci-15-00902-s001.zip › brainsci-3793649-suppl file.pdf]

Table S1. The English-language items of the Observable Social Cognition Rating Scale (OSCARS).

	None	Very Mild	Mild	Moderate	Moderately Severe	Severe	Extremely Severe
1. **Recognising other people's emotions, particularly negative emotions (sadness, fear and anger) based on facial expression, body language and/or vocal tone and rate?** EXAMPLE: When talking to someone, the individual cannot tell that the other person is upset or angry. They seem "clueless" about how other people are feeling	Can recognise strong, moderate and subtle expressions of emotions. She/he can be thought of as "socially perceptive".		Easily recognises strong and explicit expressions of emotion, such as crying, angry shouting or elated laughing. May not recognise moderately expressed emotions. However, she/he does not recognise subtle expressions, such as disappointment expressed only with a sigh or slight mouth movement.		Recognises most strong and explicit expressions. Does not recognise moderate or subtle emotional expressions		Never or does not recognise strong, moderate and subtle emotional expressions. The person must be told what emotion is being expressed ("I am very angry.")
2. **Interpreting social interactions in a malevolent, hostile manner?** EXAMPLE: The individual sees others as intending them harm, especially in ambiguous (unclear) situations. For example, she/he walks past a few people who are laughing and thinks that they are laughing at her/him. Or, the individual can't find a personal item and thinks that someone else took it. (Ask about frequency)	Individual does not immediately blame others or think that they have ill intentions towards them. She/he will consider the possibility that other factors, such as situational factors, may have caused the outcome (e.g., that others are laughing at a joke, not them).		Sometimes feels that others intend them harm that may be transient or shortlived		Frequently interprets others behaviour as ill intentioned. May sometimes accuse close acquaintances of ill will.		Widespread beliefs of ill will, which includes both close acquaintances and strangers. These beliefs are generally unfounded.
3. **Making decisions quickly (i.e., jumps to conclusions) without examining other evidence?** EXAMPLE: The individual attempts to call you and you do not answer. They immediately believe that you did not pick	Does not rush to decisions; is thoughtful and deliberate. The person seeks out other information and takes time to carefully weigh the		Sometimes uses only immediate information to make decisions. The person makes decisions using additional information some of the time and can weigh		Often uses only immediate information to make decisions. The person must be prompted or told to examine other sources of		Frequently uses only the most immediate information to make decisions. The person does not seek out additional information and seems to rush to

	up because you are mad at them. (Ask about frequency)	pros and cons before making a decision.		the pros and cons if motivated.		evidence and take their time in making decisions.		judgement almost without thinking. Seems irrational.
**4.**	**Being flexible in interpreting social situations?**<br>EXAMPLE: The individual is waiting for someone at a restaurant and they are 20 minutes late. They conclude that the person does not want meet them when in reality they may be stuck in traffic or have had a flat tire. They do not consider other alternatives for why the person is late and stick to one opinion.	When considering someone else's behaviour, she/he is able to come up with multiple reasons for why she/he acted the way they did.		Has some trouble coming up with guesses, but can do so if motivated.		Has difficulty even thinking up multiple possible explanations for others' social behaviour; Has to be prompted or asked to come up with different guesses about another person's behaviour.		Does not understand that more than one interpretation of an event is possible and is unable to generate any alternative guesses.
**5.**	**Can change or correct their interpretation of social interactions when wrong?**<br>EXAMPLE: The individual sees two people whispering and they believe they are talking about them. This belief is maintained even when told that one of the people was sharing something personal about themselves.	Is able to seek out, and weigh, evidence for and against a given belief about someone else.		The individual will consider evidence that contradicts misinterpretations she or he has made, although they might maintain the false belief anyway		The individual often avoids listening to facts that contradicts his/her views, or may argue strongly against them, and usually maintains the false belief.		The individual refuses to consider contradictory evidence. It feels impossible to talk the individual out of a belief even when the belief is clearly wrong
**6.**	**Understanding subtle jokes, sarcasm and insults in conversation?**<br>EXAMPLE: Someone states during a meal that this is best food I have ever had in a sarcastic tone and the individual does not realise that this is an insult and/or sarcasm.	The individual understands subtle jokes, insults or sarcasm.		The individual sometimes doesn't get subtle jokes or insults made by others (e.g., "Oh yes, I love working 15 hours a day!"). Seems to take longer to get the meaning of jokes and sarcasm.		The individual often does not understand subtle jokes, sarcasm, or insults and must be told what they mean.		The individual does not understand subtleties at all. Statements must be concrete and direct in order to be understood (e.g., slapstick humour).
**7.**	**Seeing things from the perspective of others (i.e., putting themselves in other people's shoes)?**<br>EXAMPLE: The individual cannot understand why someone feels upset or	Individual can be described as empathic. When watching a sad or happy film (or reading a		Seldom inquires or makes guesses about others mental states or feelings (e.g., "Do you like that?" or "Are you confused?"), but		Only reacts empathically when others express strong emotion. Does not ask about or respond to others' opinions or		Unable to accurately judge what others might be thinking or feeling, except in the most extreme circumstances

angry in a particular situation. Or, when watching a sad film, does not feel moved by it.	sad or happy book), can be moved by it.		understands when people make these statements.		experiences. When asked, may have difficulty imagining what others might be thinking.		(such as feeling sadness after the death of a loved one.)
8. **Understanding subtle social cues, hints and indirect requests (an example of an indirect request is if your son/daughter wants a toy, but rather than say so directly, comments on how pretty it is.** EXAMPLE: You are trying to read a book or watch TV and the individual keeps talking to you, even though you 970 are giving off subtle hints/signals that you are not interested in talking to them at that moment (e.g., keeping your answers short; not making eye contact).	The individual readily picks up social cues and/or indirect requests. For example, if you are busy and they start talking to you, they readily perceive that you can't speak with them at that moment.		The individual does not pick up on subtle social cues at first, but does so after a minute or two. Takes longer to process subtle cues and hints.		The individual does not pick up on social cues and it takes a number of overt cues (turning away when talking to him/her) for them to get the message.		The individual does not pick up on social cues and must be told directly. Or, the individual does not get subtle hints or indirect requests.
